# And How Would That Make You Feel? How People Expect Nudges to Influence Their Sense of Autonomy

**DOI:** 10.3389/fpsyg.2020.607894

**Published:** 2020-12-11

**Authors:** Jonas Wachner, Marieke A. Adriaanse, Denise T. D. De Ridder

**Affiliations:** Department of Social, Health & Organisational Psychology, Utrecht University, Utrecht, Netherlands

**Keywords:** nudging, autonomy, choice satisfaction, persuasion, expectations

## Abstract

**Objective:**

While nudges are increasingly utilized in public policy settings, their potential threat to autonomous choice is the topic of heated debate. Regardless of the actual effects of nudges on autonomy, the mere perception of nudges as autonomy threatening by the general public or policy makers could negatively influence nudge acceptability. The present online studies examined how people expect (different) nudges to affect their perception of autonomy.

**Methods:**

In the first study (*N* = 455), participants were presented with a hypothetical choice that employed either a default nudge, direct persuasion, or no persuasion, to steer to the desired choice. The presented influence technique was explained before participants reported their expected autonomy, as well as their expected choice satisfaction. Study 2 (*N* = 601) involved a replication of Study 1 with an additional social norm nudge condition. In Study 3 (*N* = 750), the explanation of how choice had been influenced was omitted.

**Results:**

While participants expected the default nudge to violate autonomy (Study 1), they had no such expectations for social norm nudges (Study 2). Omitting the explanation that most people are unaware of nudges influencing their choice, reduced the negative impact of nudges on expected autonomy (Study 3).

**Conclusion:**

Effects of nudges on expectations of autonomy differ by type of nudge. Negative expectations are primarily driven by the explanation that decision makers are often unaware of nudges.

## Introduction

In the past decade, nudging, generally defined as the promotion of one choice without forbidding any options or significantly changing their economic incentives (Thaler and Sunstein, 2008), has found its way into public policy applications. Nudging effectiveness has been demonstrated in a variety of domains, including dietary behavior (Arno and Thomas, 2016), saving behavior (Thaler and Benartzi, 2004), recycling (Milford et al., 2015), and many others (Benartzi et al., 2017). Despite the evidence in favor of nudges’ effectiveness, there are still other aspects of nudges that are critically debated. One key point of debate is nudges’ alleged negative impact on the decision maker’s autonomy (Bovens, 2009; House of Lords, Science, and Technology Select Committee, 2011; Hansen and Jespersen, 2013). To our knowledge, there is no empirical research done to test this allegation with a specific focus on nudges, which leaves the existence of this impact open to speculation. However, regardless of whether nudges harm autonomy, merely perceiving an attempt to change one’s decision as autonomy threatening is shown to be related to negative outcomes such as worse attitude toward the promoted issue (Pavey and Sparks, 2009), lowered autonomous motivation (Pavey and Sparks, 2009), and lowered perceived usefulness of the promoted option (Walter and Lopez, 2008). In the current study, we therefore aim to investigate people’s *expectations* of how nudges might influence their perceived autonomy (further referred to as expected autonomy), as well as related constructs. Findings from this study will give insights into how threatening people experience different kinds of nudges to be to their autonomy, decision competence and choice satisfaction, and how changes to their understanding of what a nudge is can alter the severity of the perceived threat.

Autonomy is a fundamental psychological construct that has been most prominently introduced under the realm of self-determination theory (SDT; Ryan and Deci, 2000). SDT is a highly influential psychological theory that describes three basic and universal human needs: autonomy, competence, and relatedness (Ryan and Deci, 2017). SDT posits that a person’s well-being and personal growth are dependent on the satisfaction of these three needs. This assumption has received ample empirical support, as many studies have indeed shown that satisfaction of these needs lead to positive well-being outcomes (e.g., Van den Broeck et al., 2016) and that need dissatisfaction conversely leads to negative wellbeing outcomes (e.g., Wei et al., 2005). Critical in relation to nudging, SDT also claims that autonomy is not only a determinant of well-being, but also crucial to one’s self-regulation. Specifically, according to SDT “developing a sense of autonomy and competence is critical to the processes of internalization and integration, through which a person comes to self-regulate and sustain behaviors conducive to health and well-being” (Ryan et al., 2008, p. 2).

Nudges are interventions designed to steer people’s behavior in a particular direction while preserving their freedom of choice. To achieve this objective, nudges typically make strategic use of heuristics that steer our behavior. These so-called simple “rules of thumb” guide people’s behavior without individuals being immediately aware of responding to these heuristics. For example, a study that employed the salience heuristic by rearranging the products in a kiosk (placing the fruit at the cash register, a place where clients tend to make impulsive purchases) was effective in encouraging the purchase of healthy foods (Kroese et al., 2015).

Whereas nudges may be quite promising in applying heuristics to effectively and efficiently promote desired behaviors across behavioral domains (Benartzi et al., 2017), the use of nudges also implies that behavioral choices are to some extent steered by contextual features and that individuals may not be fully aware of this. It is these undetected contextual features that, according to philosopher Bovens, undermine the decision maker’s autonomy, as the decision maker would possibly not want these features to influence their decision (Bovens, 2009). We argue that people especially expect a nudge to be harmful to autonomy when the nudge would make them unaware of certain factors and these factors’ influence on their decision.

People might have the expectation that nudges harm one’s autonomy, as an explanation of nudges generally involves that one will be subjected to subtle manipulations involving processes into which one may lack introspection, which could cause individuals to expect that this should harm their autonomy. While there have been no empirical studies on the effects of nudging on autonomy, it is evident that before doing so, a clear definition of autonomy is needed. Vugts et al. (2018) have distinguished three concepts of autonomy that have been used in the nudging literature: freedom of choice, agency, and self-constitution (Vugts et al., 2018). In the current paper we will focus on autonomy as self-constitution or being able to realize one’s personal goals and aspirations, as it puts emphasis on the person’s individuality and authenticity, and thereby taps into the aforementioned ethical concerns.

Note that people’s expectations of their sense of autonomy should not be seen as a proxy for people’s sense of autonomy had they actually been nudged, but rather as its own concept of interest. Since people are often unaware of nudges, they likely fail to reconsider their expectations of nudges once they were nudged and did not feel less autonomous, as they were not aware of being nudged in the first place. We argue that expectations, maybe more so than how participants will actually feel about their autonomy, will play an important role in people’s acceptance and judgment of nudges used in public policy. Additionally, people’s actual autonomy is again different to their sense of autonomy, however, it is not our intent to investigate or predict actual autonomy. Only people’s expectations for their sense of autonomy will be investigated.

### The Present Studies

The present study will examine whether people expect nudges to impose a threat to autonomy. We will investigate how exposure to nudges affects the subsequent expected experience of autonomy, how this in turn affects choice satisfaction, and to what degree a decreased sense of autonomy upon being nudged may be the result of an explanation of how nudges operate. Rather than manipulating actual behavior through nudges, we will use a scenario in which participants are exposed to a *hypothetical* nudge and instructed to estimate how the nudge would affect autonomy. The use of hypothetical scenarios to measure participants reaction to nudges has been employed previously (Schroeder et al., 2017), and has merit on its own, as it shows how people think of nudges and influences nudge acceptance. Every hypothetical nudge scenario will be presented together with an explanation of what a nudge is. This will ensure that people can make up their mind on nudges while having a basic understanding of how they work, similar to how people would debate nudges were they broadly implemented by public policies. We also specifically opted to not let participants make a decision in the scenario, in order to lower the risk of the occurrence of egocentric biases like the optimistic bias, which is the tendency to think one’s own risk is less than the risk of their peers (Klein and Helweg-Larsen, 2002).

In Study 1, participants were exposed to a default nudge, a direct persuasion message, or a control condition without any attempt whatsoever to influence choice. We included a direct persuasion condition to examine the effect of nudges as compared to other interventions aimed at steering a choice. We use Simons’ definition of persuasion, stating that persuasion is “human communication designed to influence the autonomous judgements and actions of others” (Simons, 2001). “Direct” in this context means that the persuasion is designed in a way that the decision maker is aware of it. Participants were asked to indicate how much pressure they experienced to comply with the promoted choice, and how much autonomy and choice satisfaction they would expect to experience. We hypothesize that participants in the control condition will experience the highest expected autonomy, highest expected satisfaction and lowest experienced pressure, whereas participants in the direct persuasion condition will experience the lowest on these outcomes and participants in the default nudge condition will report scores in between. We also hypothesize that pressure mediates the effect of condition on autonomy, and autonomy mediates the effect of condition on satisfaction.

In Study 2, we aimed to replicate and extend the effects from Study 1 by adding a second type of nudge (social proof) to test whether the effects of a default nudge generalize to other types of nudges. In Study 3, we made small alterations to the explanation of how nudges operate to explore the degree to which the various aspects of the description may account for the effects found in Study 1.

## Study 1

### Materials and Methods

#### Sample Size Estimation and Participants

For this study, an *a priori* power analysis (G^∗^Power; Faul et al., 2009) revealed a required sample size of *N* = 342 to achieve statistical power of 0.80 to detect an effect size of (η*^2^* = 0.027). This expected effect size is based on the effect size found for the difference in autonomy in an unpublished study [*N* = 140, (current authors), unpublished data] in which we used the same autonomy scale as in the current paper. Given that the pilot study was only somewhat similar to the current studies and that we wanted to have a well powered study which can reliably find smaller effects, we finally decided to increase the recommended sample size by 100, which equals 150 participants per condition, to be sure not to end up with an underpowered study.

We recruited 455 participants [61% female, mean age 37 (*SD* = 12.67; range 18–73)] through the online service Prolific. Participation was rewarded with 0.50€.

#### Design and Procedure

The present study used a one-factor between-subject design, with type of persuasion (default nudge/direct persuasion/control) as the independent variable and pressure, expected autonomy, and expected satisfaction as the main dependent variables. Participants were first told that the survey would take approximately 5 min, that they could stop participating at any time, and that their data will be anonymous and handled with care. Then, participants were presented with a scenario (Steffel et al., 2016), requiring them to imagine that they had just moved and were given the opportunity to rent “green” amenities that could reduce their electricity consumption. Next, they were presented with the list of amenities. The way of presentation varied by condition. Participants were asked to imagine themselves making a decision in this scenario.

The displayed persuasion technique was explained on the following page of the online survey. After reading the explanation, participants were asked to answer questions about their regarding pressure and their expectations for autonomy and satisfaction with their choice. Participants then had to answer an attention check, where they had to recollect what was special about the presentation of the list of amenities. Finally, we asked for demographics, asked a few explorative questions, thanked the participant, and provided the researcher’s email address in case participants had questions or remarks.

#### Scenario and Persuasion

An adjusted scenario was taken from a study by Steffel et al. (2016). All participants were asked to read the scenario carefully and imagine themselves in the scenario. The adjusted scenario read*: “You are moving into a new apartment. You are offered some ‘green’ amenities that will each add between 2 and 10$ to your monthly rent. You can see the form from which to choose the amenities, on the next page.”* On the next page, a list of 14 amenities was shown (see Supplementary Materials). In the default nudge condition, all amenities were selected by default. In the direct persuasion condition, the sentence “Please think of the environment and select as many amenities as possible!” was added. In the control condition no sentence was added and no amenities were pre-selected. Participants were not actually able to choose any amenities. After 20 s they were able to proceed to the next page. On the next page, the persuasion technique was named and explained (see Appendix A).

#### Expected Autonomy

Participants’ expected autonomy was assessed by the autonomy subscale of the Basic Psychological Needs in Exercise Scale (BPNES; Vlachopoulos and Michailidou, 2006), which in its original form measures autonomy in a physical exercise context, but was adjusted for this paper to assess autonomy in a decision making context (see Appendix B). It comprises four statements (e.g., “I feel that my choice is definitely an expression of myself.”), which participants rated on a five-point scale (“strongly disagree”—“strongly agree”). The four scores were averaged to one expected autonomy score with a good reliability (Cronbach’s α = 0.89).

#### Pressure

Pressure was measured with one single question (“How much pressure have you felt to agree to most or all green amenities?”). Participants responded on a slider with labels on the both extremes (“None at all”—“Extreme Pressure”) and the scores ranged from zero to a hundred.

#### Expected Satisfaction

Participants’ expected satisfaction with their choice was measured with the Decision Regret Scale (Brehaut et al., 2003)^1^, consisting of six statements (e.g., “It was the right decision”) which participants rated on a five-point scale (“strongly disagree”—“strongly agree”; see Appendix B). The six scores were averaged to one expected satisfaction score with a good reliability (Cronbach’s α = 0.84).

#### Attention Check

The attention check was one question that asked “what was special about the presentation of the list?” and participants had to choose the right answer out of five options. A total of 75 out of the 455 participants failed the attention check. Omitting participants who failed the attention check did not change any of the main effects. We therefore report on analyses that do include participants who failed the attention check.

#### Demographics

Finally, we asked for gender (male, female, other). As only two people answered “other,” we will omit this category from further analyses. Participants also provided their age, and answered on a slider how clear the study was to them, ranging from “I did not understand what I was supposed to do” to “Everything was clear to me.”

#### Additional Measures

We also measured expected decision making confidence with six statements, the importance of living sustainably with one question, how much they liked the persuasion technique used with one question, as well as how likely participants themselves, or others, are to be influenced by the persuasion technique they had been exposed to, with one question each. These measures will not be further discussed in this paper, but their full descriptions can be found in Supplementary Materials.

## Results

### Randomization Check

A logistic regression analysis was performed with condition as the independent variable and age, and gender as dependent variables. The results showed that participants were successfully randomized across conditions (all *ps* > 0.68).

### Descriptives and Correlation Table

Descriptives and correlations of the main variables can be found in Table 1. Participants on average reported relatively high levels of autonomy (*M* = 3.71, *SD* = 0.87) and satisfaction (*M* = 3.93, *SD* = 0.70), which were strongly correlated (*r* = 0.63, *p* < 0.001). Additionally, pressure was negatively correlated with both autonomy and satisfaction (*r* = −0.24, *p* < 0.001; *r* = −0.29, *p* < 0.001).

**TABLE 1:** Means, standard deviations, and correlations with confidence intervals.

**Variable**	***M***	***SD***	**1**	**2**	**3**
1. Pressure_1	36.89	29.90			
2. Age	37.27	12.67	−0.04		
			[−0.13, 0.05]		
3. Autonomy	3.71	0.87	−0.24**	0.13**	
			[−0.33, −0.16]	[0.04, 0.22]	
4. Satisfaction	3.93	0.70	−0.29**	0.09*	0.63**
			[−0.37, −0.20]	[0.00, 0.18]	[0.57, 0.68]

M and SD are used to represent mean and standard deviation, respectively. Values in square brackets indicate the 95% confidence interval for each correlation. The confidence interval is a plausible range of population correlations that could have caused the sample correlation (Cumming, 2014). *p < 0.05; **p < 0.01.

### Autonomy

**FIGURE 1:**
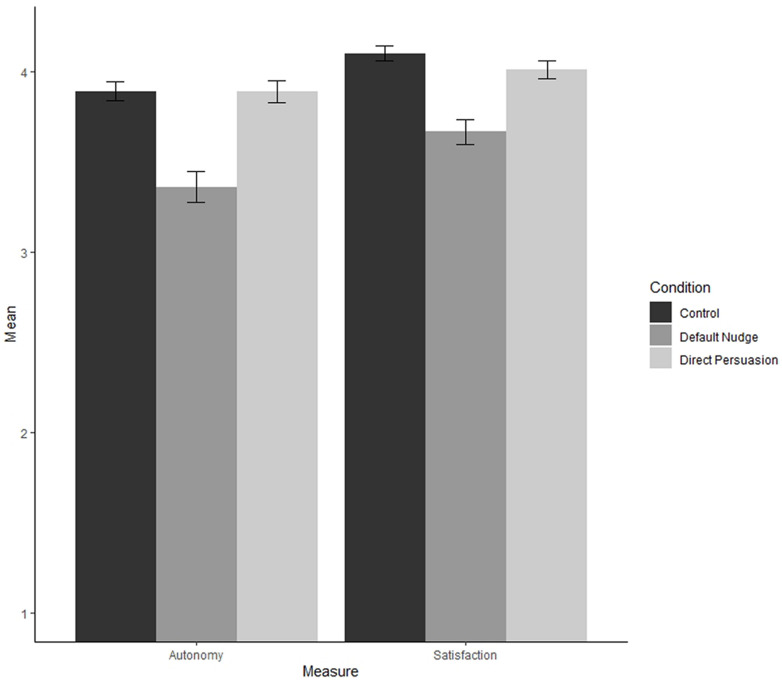
Autonomy, competence, and satisfaction per condition (Study 1).

A one-way ANOVA with condition as the independent variable and autonomy as the dependent variable revealed a significant difference of medium strength between conditions, *F*(2, 452) = 20.1, *p* < 0.001, η^2^ = 0.08 (see Figure 1). *Post-hoc* comparisons using the Tukey HSD test support part of our hypothesis, as the mean expected autonomy score for the default nudge condition (*M* = 3.36, *SD* = 1.08) was significantly lower than the mean score for the direct persuasion condition (*M* = 3.89, *SD* = 0.74, *p* < 0.001) and the control condition (*M* = 3.89, *SD* = 0.64, *p* < 0.001). Contrary to our hypothesis, the direct persuasion condition did not score lower than the control condition (*p* = 0.999)^2^.

### Pressure

A one-way ANOVA with condition as the independent variable and pressure as the dependent variable revealed a significant difference of medium strength *F*(2, 452) = 34.8, *p* < 0.001, η*^2^* = 0.13. *Post hoc* comparisons using the Tukey HSD test support our hypothesis, as the mean pressure score for the default nudge condition (*M* = 49.84, *SD* = 31.20) was significantly higher than the mean score for the direct persuasion condition (*M* = 37.58, *SD* = 28.19, *p* < 0.001), followed by the control condition (*M* = 23.27, *SD* = 23.83, *p* < 0.001).

### Satisfaction

A one-way ANOVA with condition as the independent variable and satisfaction as the dependent variable revealed a significant difference of medium strength *F*(2, 452) = 17.7, *p* < 0.001, η*^2^* = 0.07 (see Figure 1). *Post hoc* comparisons using the Tukey HSD test support part of our hypothesis, as the mean satisfaction score for the default nudge condition (*M* = 3.67, *SD* = 0.84) was significantly lower than the mean score for the direct persuasion condition (*M* = 4.01, *SD* = 0.61, *p* < 0.001) and the control condition (*M* = 4.10, *SD* = 0.54, *p* < 0.001). Contrary to our hypothesis, the direct persuasion and control condition did not differ significantly (*p* = 0.458).

Finally, we tested for serial moderation of pressure and autonomy for the effect of the nudge on expected satisfaction (further mediation models can be found in Supplementary Materials). In comparison to the control condition, the default nudge had a significant negative effect on expected satisfaction [*b* = −0.44, *t*(304) = −5.4, *p* < 0.001]. As theorized, this effect was serially mediated by pressure and expected autonomy. The indirect pathway of the effect of the nudge on expected satisfaction via pressure and expected autonomy was significant [*b*(indirect) = −0.06, *z* = −2.5, *p* = 0.011]. This pathway fully accounted for the overall impact of the nudge on expected satisfaction with the direct effect being insignificant [*b* (direct) = −0.09, *z* = −1.3, *p* = 0.183].

### Discussion

Study 1 found that participants who were exposed to a default nudge in a fictitious scenario experienced more pressure, and expected to experience less autonomy and less choice satisfaction as compared to the control and persuasion conditions. However, it is unclear whether these effects are specific to default nudges or generalize to other types of nudges. Additionally, in Study 1 the default nudge by default selected 14 options, which contrasts with common default nudges that pre-select one option over one or a few alternatives (e.g., preselection of a green energy provider over a gray energy provider). Therefore, we will replicate Study1 with a new hypothetical scenario comprising less preselected options. We will also include a second type of nudge in the design.

## Study 2

### Materials and Methods

#### Participants

As in Study 1, we again recruited 150 participants per condition, resulting in 601 participants [59% female, mean age 36 (*SD* = 11.32; range 18–78)]. Participation was rewarded with 0.70€.

#### Design and Procedure

Study 2 was designed as a conceptual replication and extension of Study 1, comprising a between-subjects design, with type of persuasion as the main independent factor (default nudge/direct persuasion/social norm nudge/control). The dependent variables were the same as in Study 1.

A few changes were made to the design: First, we added a social norm nudge condition, where participants were nudged with a description highlighting the popularity of a particular choice with the other participants. As default nudges are generally considered to exert the strongest influence on choices (Sunstein, 2016), we included a milder type of nudges to examine whether we can replicate the findings from Study 1.

Second, we implemented a more realistic scenario, where only one option could be selected. The new scenario asked participants whether they wanted 100% conventional electricity, or a mix which includes 50% green electricity at a higher price. In the default nudge condition we checked the green option by default. In the social norm nudge condition we added a sentence which stated that most tenants chose the green option. In the direct persuasion condition we added a sentence that urged participants to choose the green option. The control condition did not include any type of persuasion. Again, the used persuasion technique was explained and named to participants.

### Results

#### Randomization Check

A logistic regression was performed with condition as independent variable and age and gender as dependent variables. The results showed that participants were successfully randomized (all *ps* > 0.35).

#### Descriptives

Participants on average reported relatively high levels of autonomy (*M* = 3.66, *SD* = 0.87) and satisfaction (*M* = 3.97, *SD* = 0.73), which were strongly correlated (*r* = 0.64, *p* < 0.001). Similar to Study 1, pressure was negatively correlated with both autonomy and satisfaction (*r* = −0.28, *p* < 0.001; *r* = −0.42, *p* < 0.001).

#### Autonomy

**FIGURE 2:**
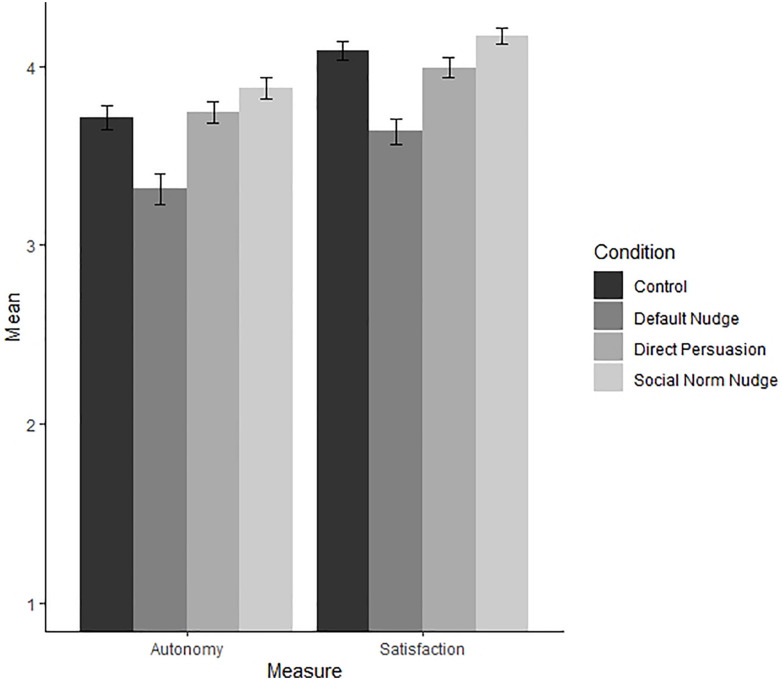
Autonomy, competence, and satisfaction per condition (Study 2).

A one-way ANOVA with condition as the independent variable and autonomy as the dependent variable revealed a significant difference of medium strength *F*(3, 597) = 12.7, *p* < 0.001, η^2^ = 0.06 (see Figure 2). *Post hoc* comparisons using the Tukey HSD test support part of our hypothesis, as the mean autonomy score for the default nudge condition (*M* = 3.31, *SD* = 1.02) was significantly lower than the mean score for the direct persuasion condition (*M* = 3.74, *SD* = 0.74, *p* < 0.001), the social norm nudge condition (*M* = 3.88, *SD* = 0.74, *p* < 0.001), and the control condition (*M* = 3.72, *SD* = 0.84, *p* < 0.001). The direct persuasion, social norm nudge, and control condition did not differ significantly (all *ps* > 0.31).

#### Pressure

A one-way ANOVA with condition as the independent variable and pressure as the dependent variable revealed a significant difference of medium strength *F*(3, 597) = 14.3, *p* < 0.001, η^2^ = 0.07. *Post hoc* comparisons using the Tukey HSD test support part of our hypothesis, as the mean pressure score for the default nudge condition (*M* = 47.80, *SD* = 32.39) was significantly higher than the mean score for the control condition (*M* = 27.14, *SD* = 24.87, *p* < 0.001) and the social norm nudge condition (*M* = 35.27, *SD* = 29.36, *p* = 0.002). Contrary to our hypothesis, the direct persuasion condition (*M* = 44.52, *SD* = 30.69) did not differ from the default nudge condition (*p* = 0.787). Yet, in line with our hypothesis, the social norm nudge condition scored also significantly lower than the direct persuasion condition (*p* = 0.044). Finally, the social norm nudge condition scored marginally lower on pressure compared to the control condition (*p* = 0.093), while the direct persuasion condition and control condition did not differ (*p* = 0.787).

#### Satisfaction

A one-way ANOVA with condition as the independent variable and satisfaction as the dependent variable revealed a significant difference of medium strength *F*(3, 597) = 16.6, *p* < 0.001, η^2^ = 0.08 (see Figure 2). *Post hoc* comparisons using the Tukey HSD test support part of our hypothesis, as the mean satisfaction score for the default nudge condition (*M* = 3.64, *SD* = 0.88) was significantly lower than the mean score for the direct persuasion condition (*M* = 3.99, *SD* = 0.68, *p* < 0.001), the social norm nudge condition (*M* = 4.17, *SD* = 0.58, *p* < 0.001) and the control condition (*M* = 4.09, *SD* = 0.66, *p* < 0.001). Contrary to our hypothesis, the direct persuasion and control condition did not differ significantly (*p* = 0.638). The direct persuasion and social norm nudge condition did not differ either (*p* = 0.124).

Finally, we tested for serial moderation of pressure and autonomy for the effect of the nudge on expected satisfaction (further mediation models can be found in Supplementary Materials). Compared to the control condition, the default nudge condition had a significant negative effect on expected satisfaction [*b* = −0.45, *t*(301) = −5.1, *p* < 0.001]. This effect was partially mediated by pressure and expected autonomy. The indirect pathway of the effect of the nudge on expected satisfaction via pressure and expected autonomy was significant [*b*(indirect) = −0.09, *z* = −3.7, *p* < 0.001]. This pathway partially accounted for the overall impact of the nudge on expected satisfaction, with the direct effect being smaller, however, still significant [*b* (direct) = −0.16, *z* = −2.2, *p* = 0.025].

### Discussion

Study 2 replicated the findings for the default nudge within a new decision scenario using a more conventional default nudge, comprising a lower number of preselected options. Study 2 also aimed to test whether the effects of default nudges found in Study 1 extended to other types of nudges. Findings indicate that participants did not consider the social norm nudge as autonomy threatening and even less so than the direct persuasion message, supporting the idea that different kinds of nudges differ in how autonomy threatening they appear. Study 3 was designed to test whether this difference between the social proof and default nudge could be replicated. We examined the extent to which the descriptions of the nudges being used were driving experienced pressure, and the expectations of autonomy and satisfaction, to disentangle nudge exposure from nudge explanation. We therefore included a condition in which the explanation, that nudges are mostly unnoticed by the decision maker, was omitted.

## Study 3

### Materials and Methods

#### Participants

Similar to Study 1 and Study 2, we tested 150 participants per condition, resulting in 750 participants [60% female, mean age 34 (SD = 12.01; range 18–81)]. Participation was rewarded with 0.50€.

#### Design and Procedure

The study was similar to Study 2 in terms of the general procedure but involved different experimental conditions. Study 3 included one control condition and four experimental conditions. The experimental conditions involved either a default or a social norm nudge, and either nudge was followed by a description highlighting its goal, its working mechanism, and its name (“nudge”). The description either included or omitted the statement that most nudges are not noticed by the decision maker. The control condition was similar to control conditions in Study 1 and Study 2.

### Results

#### Randomization Check

A logistic regression was performed with conditions as independent variable and age and gender as dependent variables. The results showed that participants were successfully randomized (all *ps* > 0.08).

#### Descriptives

Participants on average reported relatively high levels of autonomy (*M* = 3.61, *SD* = 0.94) and satisfaction (*M* = 3.91, *SD* = 0.81), which were strongly correlated (*r* = 0.60, *p* < 0.001). Additionally, pressure was negatively correlated with both autonomy and satisfaction (*r* = −0.30, *p* < 0.001; *r* = −0.46, *p* < 0.001).

#### Autonomy

**FIGURE 3:**
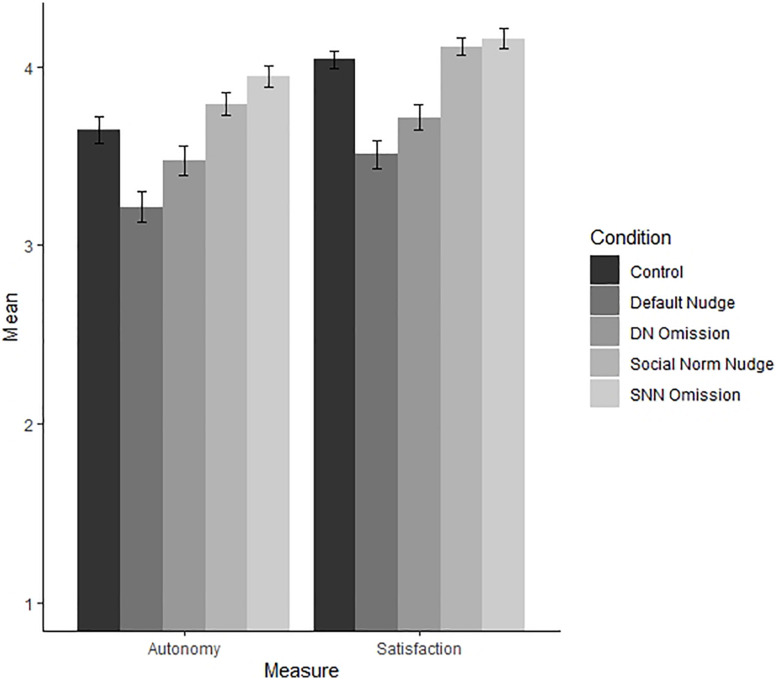
Autonomy, competence, and satisfaction per condition (Study 3).

A one-way ANOVA with condition as the independent variable and autonomy as the dependent variable revealed a significant difference of medium strength [*F*(4, 745) = 14.6, *p* < 0.001, η^2^ = 0.07; see Figure 3]. *Post hoc* comparisons using the Tukey HSD test indicated that compared to the control condition (*M* = 3.65, *SD* = 0.93), the default nudge condition scored significantly lower (*M* = 3.22, *SD* = 1.05, *p* < 0.001), while the default nudge omission condition did not score significantly different (*M* = 3.48, *SD* = 0.99, *p* = 0.409). The social norm nudge condition did not score significantly different (*M* = 3.79, *SD* = 0.78, *p* = 0.645) from the control condition and the social norm nudge omission condition even scored significantly higher (*M* = 3.94, *SD* = 0.74, *p* = 0.037) than the control condition.

The default nudge condition did not score significantly different to the default nudge omission condition (*p* = 0.094), and the social norm nudge condition did not score significantly different to the social norm nudge omission condition (*p* = 0.582).

#### Pressure

A one-way ANOVA with condition as the independent variable and Pressure to take as many amenities as possible as the dependent variable revealed a significant difference of small strength [*F*(4, 745) = 6.69. *p* < 0.001, η^2^ = 0.03]. *Post hoc* comparisons using the Tukey HSD test indicated that participants in the default nudge condition (*M* = 46.82, *SD* = 33.52) scored significantly higher on pressure than participants in the control condition (*M* = 35.32, *SD* = 30.30, *p* = 0.013), the social norm nudge condition (*M* = 34.68, *SD* = 28.94, *p* = 0.007) and the social norm nudge omission condition (*M* = 32.58, *SD* = 30.18, *p* < 0.001, *p* < 0.001). The default nudge omission condition (*M* = 45.16, *SD* = 32.50), opposed to the default nudge condition, scored only marginally higher on pressure than the control condition (*p* = 0.052). Still, the default nudge omission condition scored significantly lower than the social norm nudge (*p* = 0.031) and social norm nudge omission condition (*p* = 0.005). Contradicting our hypothesis, the omission conditions did not differ significantly from the non-omission conditions (*ps* > 0.977).

#### Satisfaction

A one-way ANOVA with condition as the independent variable and choice satisfaction as the dependent variable revealed a significant difference of medium strength [*F*(4, 745) = 12.0. *p* < 0.001, η^2^ = 0.10; see Figure 3]. *Post hoc* comparisons using the Tukey HSD test indicated that participants in the default nudge condition (*M* = 3.51, *SD* = 0.99) scored significantly lower on satisfaction as participants in the control condition (*M* = 4.04, *SD* = 0.57, *p* < 0.001), the social norm nudge condition (*M* = 4.12, *SD* = 0.63, *p* < 0.001), and the social norm nudge omission condition (*M* = 4.16, *SD* = 0.69, *p* < 0.001). The default nudge omission condition (*M* = 3.72, *SD* = 0.88) also did score lower on satisfaction compared to the social norm nudge (*p* < 0.001) and social norm nudge omission condition (*p* < 0.001). Contradicting our hypothesis, the default nudge condition and the default nudge omission condition did not differ significantly (*p* = 0.142). Finally, opposed to autonomy, participants in the default nudge omission condition also scored significantly lower on satisfaction compared to the control condition (*p* = 0.003).

Finally, we tested for serial moderation of both pressure and autonomy for the effect of the default nudge without an explanation on expected satisfaction (further mediation models can be found in Supplementary Materials). In comparison to the control condition, the default nudge condition negatively affected expected satisfaction [*b* = −0.53, *t*(299) = −5.7, *p* < 0.001]. This effect was, however, not mediated by pressure and expected autonomy. The indirect pathway of the effect of the nudge on expected satisfaction via pressure and expected autonomy was significant [*b*(indirect) = −0.02, *z* = −2.2, *p* = 0.031]. This pathway partially accounted for the overall impact of the nudge on expected satisfaction [*b* (direct) = −0.27, *z* = −3.6, *p* < 0.001].

### Discussion

Study 3 replicated the findings of Studies 1 and 2 with regard to the two nudge conditions using the explanation including the section on awareness: they were again seen as more negative in its effect on pressure, autonomy and satisfaction. Moreover, similar to Studies 1 and 2, pressure mediated the effect of condition on autonomy, and autonomy mediated the effect of condition on satisfaction. The social norm nudge condition scored the same as the control condition on the three dependent variables, similar to Study 2. Study 3 also tested whether the effects of the nudges on pressure, autonomy and satisfaction were driven by the description of the nudge. Importantly, omitting the explanation regarding awareness improved expected autonomy to the extent that the default nudge condition was now no longer statistically different to the control group. Omitting the awareness-sentence from the social norm nudge description even resulted in *higher* expected autonomy compared to the control condition, suggesting that people consider some nudges as autonomy supportive.

## General Discussion

The goal of the current series of studies was to investigate people’s expectations of the effects of nudges on autonomy. This was done with hypothetical nudges that were explained to the participants, so that participants’ expectations of autonomy and other measures reflect the expectations and opinions that people have when they discuss nudging, such as in settings where the public policy or interventions are debated (e.g., private discussion, discussion of company policies, political discussion). Additionally, we tested whether experienced pressure mediates the effects of the persuasion techniques on expected autonomy, and whether expected autonomy in turn predicts participants expected satisfaction with their choice.

First, all three studies show that participants who were confronted with a hypothetical default nudge anticipated lower scores of autonomy and choice satisfaction and reported higher scores of pressure, compared to participants confronted with either direct persuasion, social norm nudges, or no persuasion at all. These predictions by lay people are similar to those of philosophers, who also suspect nudges to negatively affect autonomy (Bovens, 2009; Hansen and Jespersen, 2013). Second, we consistently found that social norm nudges do not lead to more negative expectations of autonomy, compared to the control and direct persuasion conditions. This suggests that different types of nudges are seen as differently affecting autonomy and effects of one nudge cannot be generalized to all nudges. A possible explanation for the absence of a negative effect of the social norm nudge on expected autonomy might be that it is seen as less intrusive than a default nudge,. Also, the fact that other people choose a certain option might be seen as valid persuasion, as especially descriptive norms are generally experienced as an implicit recommendation. Future research should include a wider range of nudges before strong conclusions can be drawn on the impact of nudges on anticipated autonomy. Future research should also examine in what way perceptions of default nudges differ from social norm nudges and other types of nudges to identify what element of nudges may be harmful to expected autonomy.

Third, in Study 3 we found that participants in the nudge conditions had similar expectations for autonomy and satisfaction compared to the control condition when they were not told that nudges usually work without the decision maker’s awareness. However, this omission did not affect participants’ experienced pressure. This indicates that the processes by which the knowledge of nudges’ covert nature affect pressure and anticipated autonomy are at least partially independent. Moreover, this finding also suggests that transparency about nudging may not necessarily resolve ethical issues about nudging, as an explanation of how nudges operate may increase feelings of worry rather than decrease them. Indeed, it has been pointed out that disclosure is not a panacea for nudge legitimacy (Loewenstein et al., 2014).

Furthermore, we found that effects of persuasion techniques on expected satisfaction can be partially explained by autonomy. This is in line with the literature on autonomy, which has documented that autonomy is a predictor of different kinds of general satisfaction and happiness outcomes (e.g., Finn, 2001; Howell et al., 2011). This finding suggests that when people expect a nudge to harm their feeling of autonomy, this could lead to the expectation to be less satisfied with one’s choice—although our studies do not allow for establishing causality. Still, these findings illustrate the relevance of understanding nudges’ effect on autonomy, as the association with choice satisfaction will likely influence future choices. This is important for understanding the long-term effectiveness of nudging interventions.

Additionally, the data confirmed our hypothesis that experienced pressure partially explains the relation between a persuasion technique and expected autonomy. This suggests that participants who experience more pressure expect to experience less autonomy. This relation between pressure and autonomy is in line with previous research, where it was found that controlling environments, as opposed to supporting environments, are harmful to autonomy and well-being (Gagne, 2003; Adie et al., 2012). Again, it has to be noted that we cannot make claims of causality. Still, as our studies showed that it depends on the type of nudge how much pressure people will experience, we demonstrated that a careful selection of a nudge may lead to stronger feelings of autonomy, which in turn could result in a higher satisfaction with one’s choice.

The current set of studies showed that, some nudges are expected to have a negative effect on autonomy. However, both the type of nudge and the understanding of what a nudge is, are crucial to this impact. Future research should investigate what aspects of a nudge and people’s understanding of a nudge makes them appear threatening to autonomy, in order to design nudges that are not only as effective as possible, but also as nonthreatening as possible.

## Data Availability Statement

The raw data supporting the conclusions of this article will be made available by the authors, without undue reservation, to any qualified researcher.

## Ethics Statement

The studies involving human participants were reviewed and approved by the Peter van der Heijden, The Board of the Faculty of Social and Behavioral Sciences Utrecht University. The patients/participants provided their written informed consent to participate in this study.

## Author Contributions

JW was the main author of the manuscript and conducted the experiments. MA and DD together with JW designed the experiments and regularly gave feedback on drafts of the manuscript. All authors contributed to the article and approved the submitted version.

## Conflict of Interest

The authors declare that the research was conducted in the absence of any commercial or financial relationships that could be construed as a potential conflict of interest.

## Figures and Tables

**FIGURE B1:**
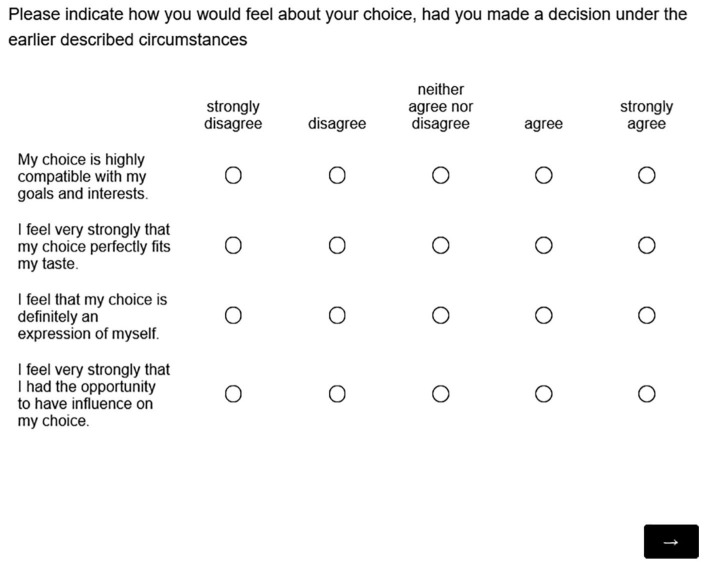
Expected autonomy questionnaire.

**FIGURE B2:**
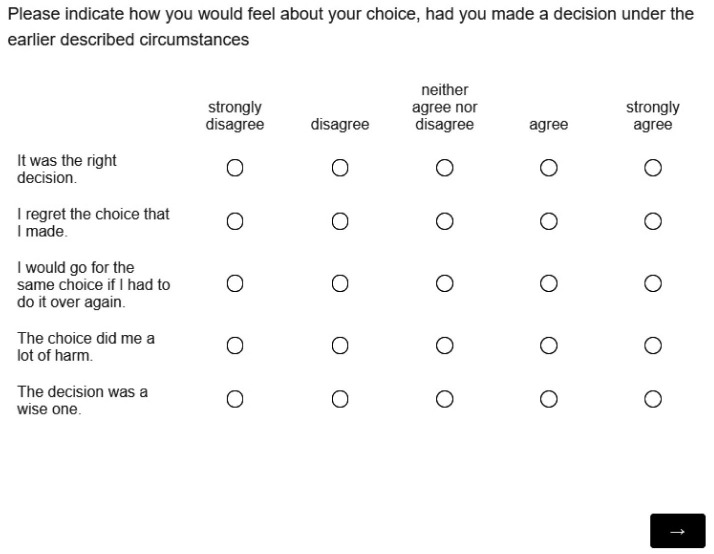
Expected satisfaction questionnaire. Question two and four are reversed.

## Footnotes

1 We decided to use this scale as it is formulated in such a broad way that it can be used in our study. Even though there are questionnaires which aim to specifically measure satisfaction, these questionnaires are specifically tailored to shared decision making in medical settings (Satisfaction With Decision scale; Wills and Holmes-Rovner, 2003) or decisions that will impact behavior for long periods of time (Sainfort and Booske, 2000). The Decision Regret Scale was found to be strongly negatively correlated with the Satisfaction With Decision scale (Brehaut et al., 2003) and will be used in our study as a mirrored proxy for satisfaction with one’s choice.

2 Autonomy, competence and satisfaction are not normally distributed. Following Tabachnick and Fidell (2007), 20 or more degrees of freedom are necessary if the data is not normally distributed. Due to the high number of participants, this is given for all our analyses. The same variables also have heteroscedasticity of variance. Here, Tabachnik, and Fidell argue that an alpha of 0.025 or 0.01 should be used, which does mean that the effect for autonomy, competence, and satisfaction are still significant.

## Appendix A

### Descriptions of the Manipulations

Description of the default nudge: As you may have noticed, all boxes are checked by default. This is done to increase the number of amenities that will be chosen. By having all the boxes ticked by default, people will deselect the options they definitely do not want but keep options they are uncertain about selected. Also, having all the boxes ticked by default will give the impression that it is normal to select many amenities. This technique is called Nudging. It is most often used without the decision maker being aware of it.

Description of the direct persuasion: As you may have noticed, above the list was a message that said “Please think of the environment and select as many amenities as possible!.” This is done to increase the number of amenities that will be chosen. By directly talking to the people and reminding them of the environment, more boxes will be selected. This technique is called direct persuasion.

Description of the control condition: As you may have noticed, the options were presented in an unbiased manner. This is done so the way in which the form is presented does not influence your decision.

## References

[B1] Adie J. W. Duda J. L. Ntoumanis N. (2012). Perceived coach-autonomy support, basic need satisfaction and the well-and ill-being of elite youth soccer players: a longitudinal investigation. *Psychol. Sport Exerc.* 13 51–59. 10.1016/j.psychsport.2011.07.008

[B2] Arno A. Thomas S. (2016). The efficacy of nudge theory strategies in influencing adult dietary behaviour: a systematic review and meta-analysis. *BMC Public Health* 16:676. 10.1186/s12889-016-3272-x 27475752 PMC4967524

[B3] Benartzi S. Beshears J. Milkman K. L. Sunstein C. R. Thaler R. H. Shankar M. (2017). Should governments invest more in nudging? *Psychol. Sci.* 28 1041–1055. 10.1177/0956797617702501 28581899 PMC5549818

[B4] Bovens L. (2009). “The ethics of nudge,” in *Preference Change Theory and Decision Library*, Vol. 42 eds Grüne-Yanoff T. Hansson S. O. (Dordrecht: Springer), 207–219. 10.1007/978-90-481-2593-7_10

[B5] Brehaut J. C. O’Connor A. M. Wood T. J. Hack T. F. Siminoff L. Gordon E. (2003). Validation of a decision regret scale. *Med. Decis. Mak.* 23 281–292. 10.1177/0272989X03256005 12926578

[B6] Cumming G. (2014). The new statistics: why and how. *Psychol. Sci.* 25 7–29. 10.1177/0956797613504966 24220629

[B7] Faul F. Erdfelder E. Buchner A. Lang A.-G. (2009). Statistical power analyses using G^∗^Power 3.1*:* tests for correlation and regression analyses. *Behav. Res. Methods* 41 1149–1160. 10.3758/brm.41.4.1149 19897823

[B8] Finn C. P. (2001). Autonomy: an important component for nurses’ job satisfaction. *Int. J. Nurs. Stud.* 38 349–357. 10.1016/s0020-7489(00)00065-1 11245871

[B9] Gagne M. (2003). Autonomy support and need satisfaction in the motivation and well-being of gymnasts. *J. Appl. Sport Psychol.* 15 372–390. 10.1080/714044203

[B10] Hansen P. G. Jespersen A. M. (2013). Nudge and the manipulation of choice: a framework for the responsible use of the nudge approach to behaviour change in public policy. *Eur. J. Risk Regul.* 4 3–28. 10.1017/s1867299x00002762

[B11] House of Lords, Science, and Technology Select Committee (2011). *Behaviour Change: Report.* London: Authority of the House of Lords.

[B12] Howell R. T. Chenot D. Hill G. Howell C. J. (2011). Momentary happiness: the role of psychological need satisfaction. *J. Happiness Stud.* 12 1–15. 10.1007/s10902-009-9166-1

[B13] Klein C. T. Helweg-Larsen M. (2002). Perceived control and the optimistic bias: a meta-analytic review. *Psychol. Health* 17 437–446. 10.1080/0887044022000004920

[B14] Kroese F. M. Marchiori D. R. de Ridder D. T. (2015). Nudging healthy food choices: a field experiment at the train station. *J. Public Health* 38 e133–e137. 10.1093/pubmed/fdv096 26186924

[B15] Loewenstein G. Sunstein C. R. Golman R. (2014). Disclosure: psychology changes everything. *Annu. Rev. Econom.* 6, 391–419. 10.1146/annurev-economics-080213-041341

[B16] Milford A. B. Øvrum A. Helgesen H. (2015). *Nudges to Increase Recycling and Reduce Waste.* Norwegian Agricultural Economics Research Institute Available online at: http://nilf.no/publikasjoner/Discussion_Papers/2015/dp-2015-01.pdf (accessed November 29, 2020).

[B17] Pavey L. Sparks P. (2009). Reactance, autonomy and paths to persuasion: examining perceptions of threats to freedom and informational value. *Motiv. Emot.* 33 277–290. 10.1007/s11031-009-9137-1

[B18] Ryan R. M. Deci E. L. (2000). Self-determination theory and the facilitation of intrinsic motivation, social development, and well-being. *Am. Psychol.* 55 68–78. 10.1037/0003-066x.55.1.68 11392867

[B19] Ryan R. M. Deci E. L. (2017). *Self-Determination Theory: Basic Psychological Needs in Motivation, Development, and Wellness*, 1 Edn New York, NY: The Guilford Press.

[B20] Ryan R. M. Patrick H. Deci E. L. Williams G. C. (2008). Facilitating health behaviour change and its maintenance: interventions based on self-determination theory. *Eur. Health Psychol.* 10 2–5. 10.1521/978.14625/28806

[B21] Sainfort F. Booske B. C. (2000). Measuring post-decision satisfaction. *Med. Decis. Mak.* 20 51–61. 10.1177/0272989X0002000107 10638537

[B22] Schroeder J. Waytz A. Epley N. (2017). Endorsing help for others that you oppose for yourself: mind perception alters the perceived effectiveness of paternalism. *J. Exp. Psychol. Gen.* 146 1106–1125. 10.1037/xge0000320 28557510

[B23] Simons H. W. (2001). *Persuasion in Society.* Thousand Oaks, CA: Sage Publications.

[B24] Steffel M. Williams E. F. Pogacar R. (2016). Ethically deployed defaults: transparency and consumer protection through disclosure and preference articulation. *J. Mark. Res.* 53 865–880. 10.1509/jmr.14.0421 11670861

[B25] Sunstein C. R. (2016). People prefer system 2 nudges (kind of). *Duke Law J.* 66:121. 10.2139/ssrn.2731868 32864128

[B26] Tabachnick B. G. Fidell L. S. (2007). *Using Multivariate Statistics.* Boston, MA: Pearson/Allyn & Bacon.

[B27] Thaler R. Sunstein C. (2008). *Nudge: Improving Decisions about Health, Wealth, and Happiness.* New Haven, CT: Yale University Press.

[B28] Thaler R. H. Benartzi S. (2004). Save more tomorrow: using behavioral economics to increase employee saving. *J. Polit. Econ.* 112 S164–S187. 10.1086/380085

[B29] Van den Broeck A. Ferris D. L. Chang C. H. Rosen C. C. (2016). A review of self-determination theory’s basic psychological needs at work. *J. Manag.* 42 1195–1229. 10.1177/0149206316632058

[B30] Vlachopoulos S. P. Michailidou S. (2006). Development and initial validation of a measure of autonomy, competence, and relatedness in exercise: the basic psychological needs in exercise scale. *Meas. Phys. Educ. Exerc. Sci.* 10 179–201. 10.1207/s15327841mpee1003_4

[B31] Vugts A. van den Hoven M. De Vet E. Verweij M. (2018). How autonomy is understood in discussions on the ethics of nudging. *Behav. Public Policy* 4, 1–16. 10.1017/bpp.2018.5

[B32] Walter Z. Lopez M. S. (2008). Physician acceptance of information technologies: role of perceived threat to professional autonomy. *Decis. Support Syst.* 46 206–215. 10.1016/j.dss.2008.06.004

[B33] Wei M. Shaffer P. A. Young S. K. Zakalik R. A. (2005). Adult attachment, shame, depression, and loneliness: the mediation role of basic psychological needs satisfaction. *J. Couns. Psychol.* 52 591–601. 10.1037/0022-0167.52.4.591

[B34] Wills C. E. Holmes-Rovner M. (2003). Patient comprehension of information for shared treatment decision making: state of the art and future directions. *Patient Educ. Couns.* 50 285–290. 10.1016/s0738-3991(03)00051-x 12900101

