# Supplementary material for: And How Would That Make You Feel? How People Expect Nudges to Influence Their Sense of Autonomy
Source: Front Psychol. 2020 Dec 11;11:607894. doi: 10.3389/fpsyg.2020.607894 (PMC7759476; doi:10.3389/fpsyg.2020.607894)
Supplement: Supplementary file 1 [file Data_Sheet_1.PDF]

# And How Would That Make You Feel?
# How People Expect Nudges to Influence Their Sense of Autonomy

## Additional Mediation Analyses Study 1

We used mediation analysis (MacKinnon, Fairchild, & Fritz, 2007) to test whether experienced pressure explains the effect of condition (nudge versus control) on autonomy. As can be seen in Figure 1, pressure partially mediated the effect of condition on autonomy.

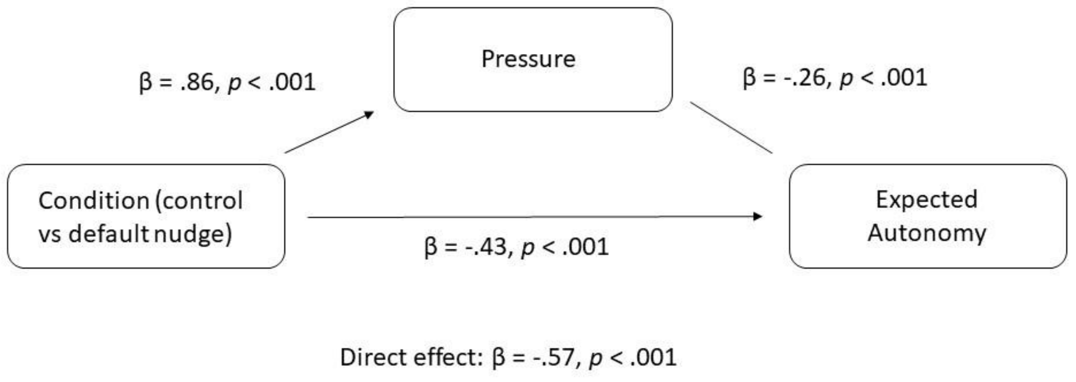


*Figure 1.* Mediation of Pressure of the Effect of Condition (control vs default nudge condition) on Expected Autonomy in Study 1.

We also used mediation analysis to test whether autonomy mediates the effect of condition (control vs default nudge) on satisfaction. As can be seen in Figure 2, autonomy partially mediated the relation between condition and expected satisfaction.

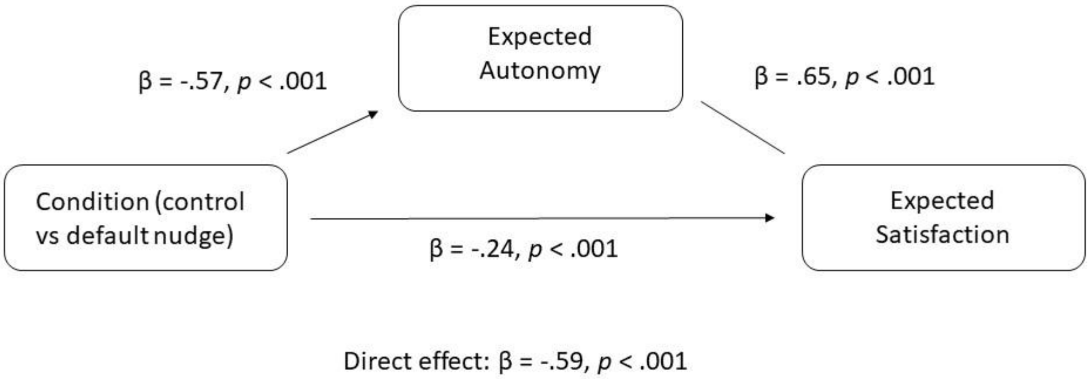


*Figure 2.* Mediation of Expected Autonomy of the Effect of Condition (control vs default nudge condition) on Expected Autonomy in Study 1.

**Additional Mediation Analyses Study 2**

We then used mediation analysis to test whether pressure mediates the effect of condition (control vs default nudge) on expected autonomy. As can be seen in Figure 3, pressure partially mediated the effect of condition on expected autonomy.

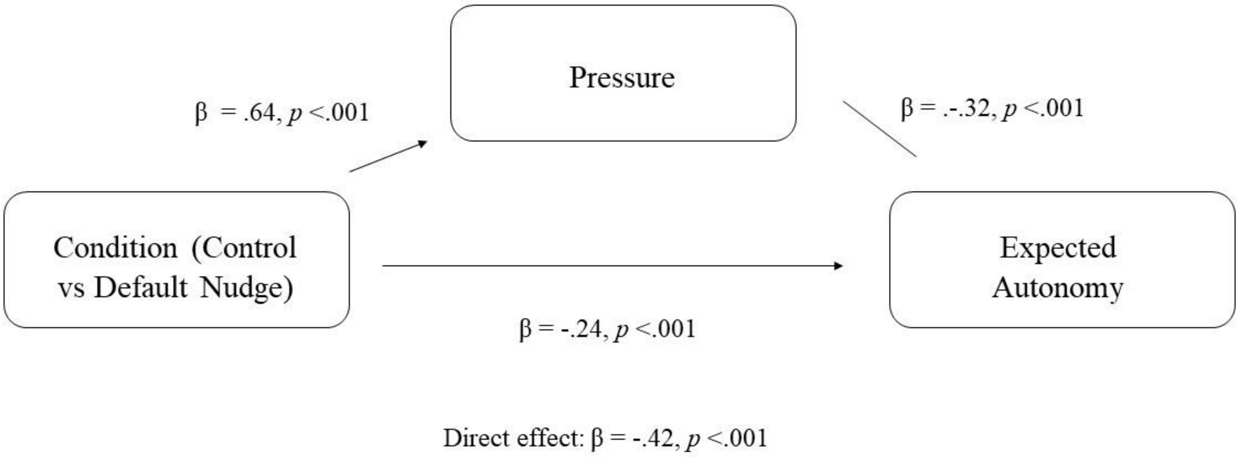


*Figure 3.* Mediation of Pressure of the Effect of Condition (control vs default nudge

condition) on Expected Autonomy in Study 2.

We also used mediation analysis to test whether pressure mediates the effect of condition (default nudge vs social norm nudge) on autonomy. As can be seen in Figure 4, pressure partially mediated the effect of condition on expected autonomy.

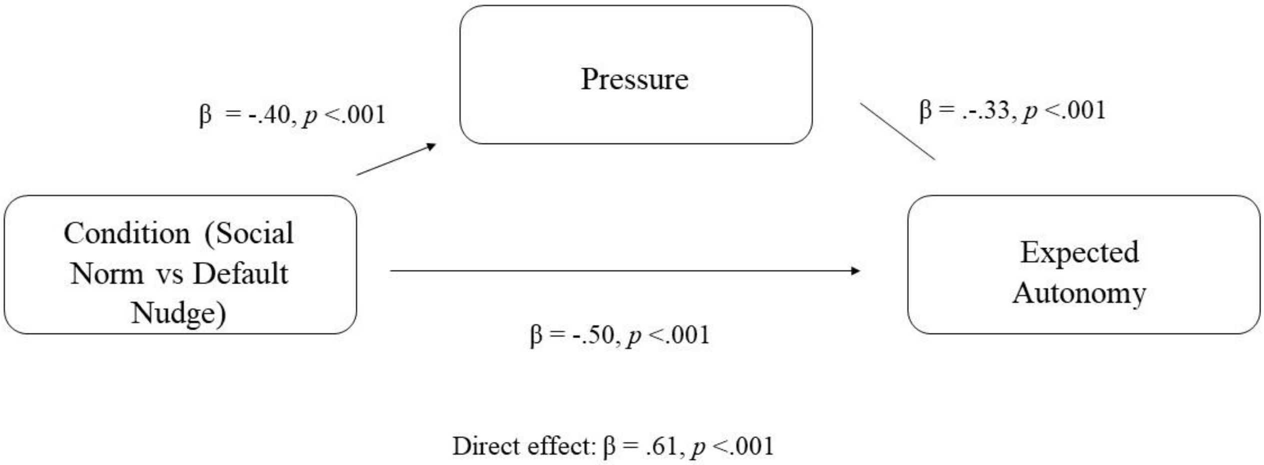


*Figure 4.* Mediation of Pressure of the Effect of Condition (social norm nudge vs default nudge condition) on Expected Autonomy in Study 2.

We then used mediation analysis to test whether autonomy mediates the effect of condition (control vs default nudge) on satisfaction. As can be seen in Figure 5, expected autonomy partially mediated the effect of condition on expected satisfaction.

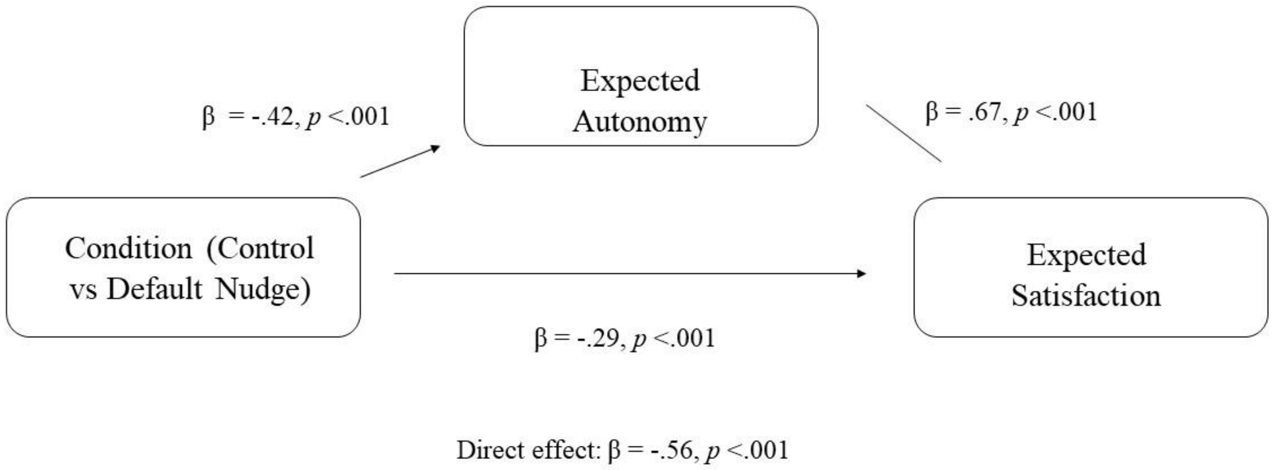


*Figure 5.* Mediation of Expected Autonomy of the Effect of Condition (control vs default nudge condition) on Expected Satisfaction in Study 2.

We also used mediation analysis to test whether autonomy mediates the effect of condition (default nudge vs social norm nudge) on satisfaction. As can be seen in Figure 6, expected autonomy partially mediated the effect of condition on expected satisfaction.

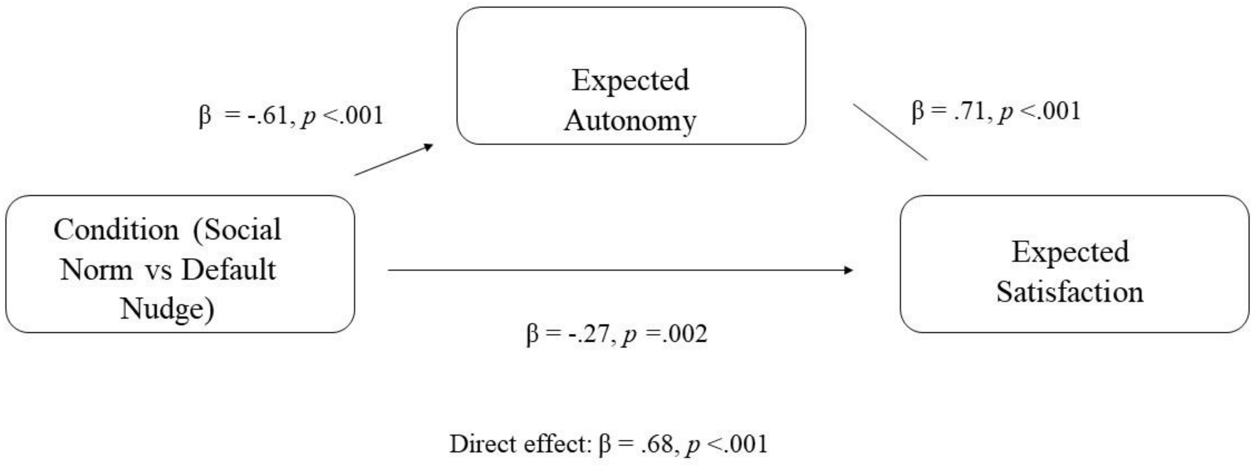


*Figure 6.* Mediation of Expected Autonomy of the Effect of Condition (social norm nudge vs default nudge condition) on Expected Satisfaction in Study 2.

Additional Mediation Analyses Study 3

We then used mediation analysis to test whether pressure mediates the effect of condition (control vs default nudge) on autonomy. As can be seen in Figure 7, pressure partially mediated the effect of condition on expected autonomy.

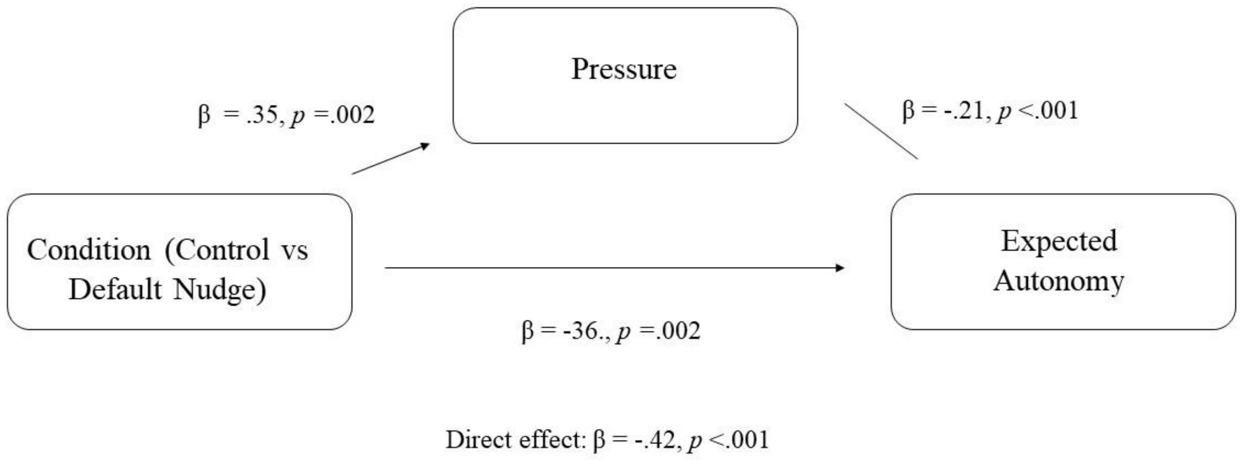


*Figure 7*. Mediation of Pressure of the Effect of Condition (control vs default nudge condition) on Expected Autonomy in Study 3.

We also used mediation analysis to test whether pressure mediates the effect of condition (default nudge vs social norm nudge) on autonomy. As can be seen in Figure 8, pressure partially mediated the effect of condition on expected autonomy.

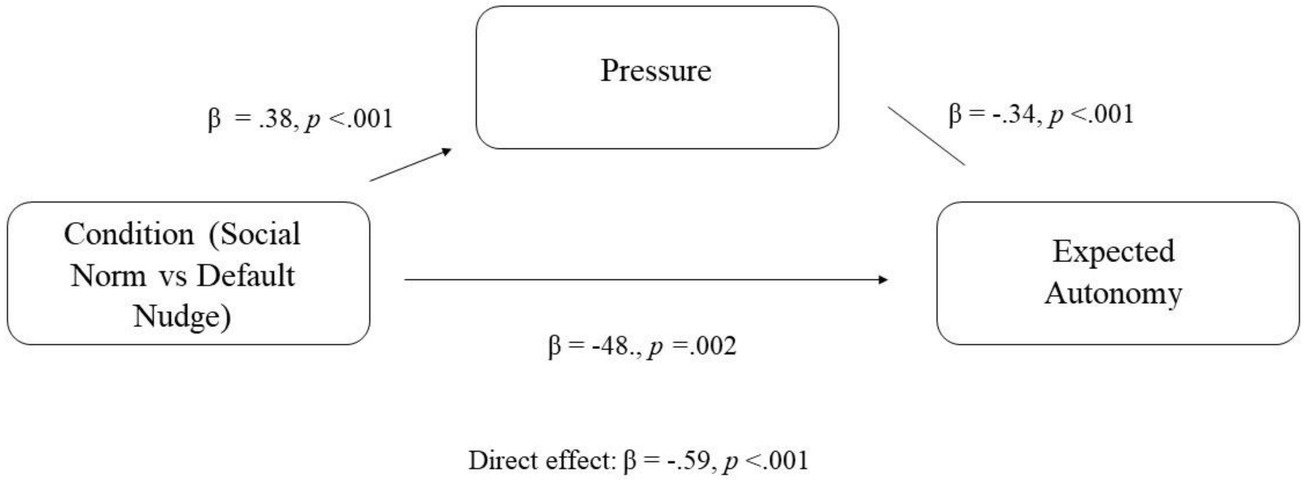


*Figure 8.* Mediation of Pressure of the Effect of Condition (social norm nudge vs default nudge condition) on Expected Autonomy in Study 3.

We then used mediation analysis to test whether autonomy mediates the effect of condition (control vs default nudge) on satisfaction. As can be seen in Figure 9, expected autonomy partially mediated the effect of condition on expected satisfaction.

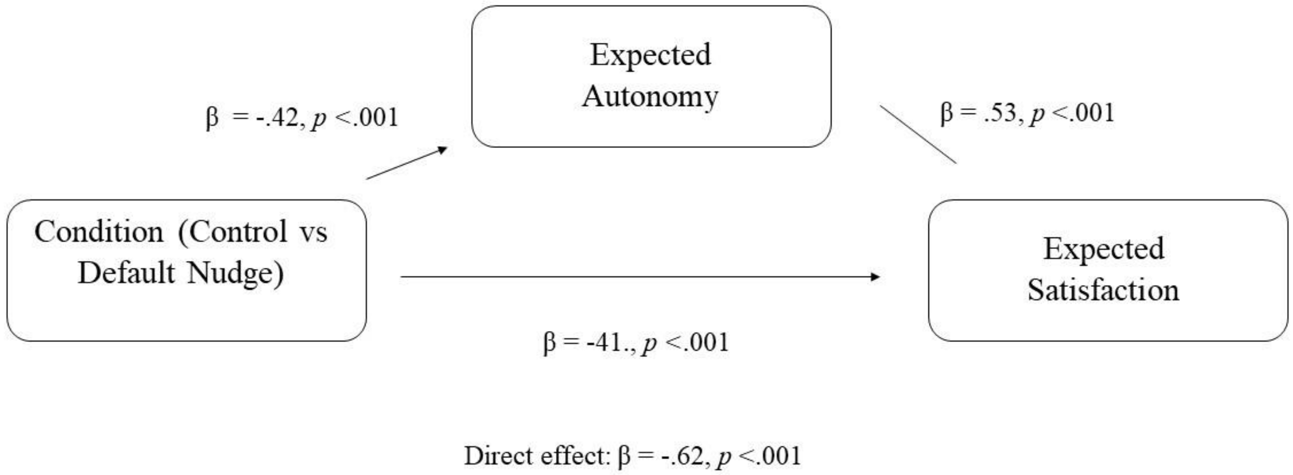


*Figure 9.* Mediation of Expected Autonomy of the Effect of Condition (control vs default nudge condition) on Expected Satisfaction in Study 3.

We then used mediation analysis to test whether autonomy mediates the effect of condition (default nudge vs social norm nudge) on satisfaction. As can be seen in Figure 10, expected autonomy partially mediated the effect of condition on expected satisfaction.

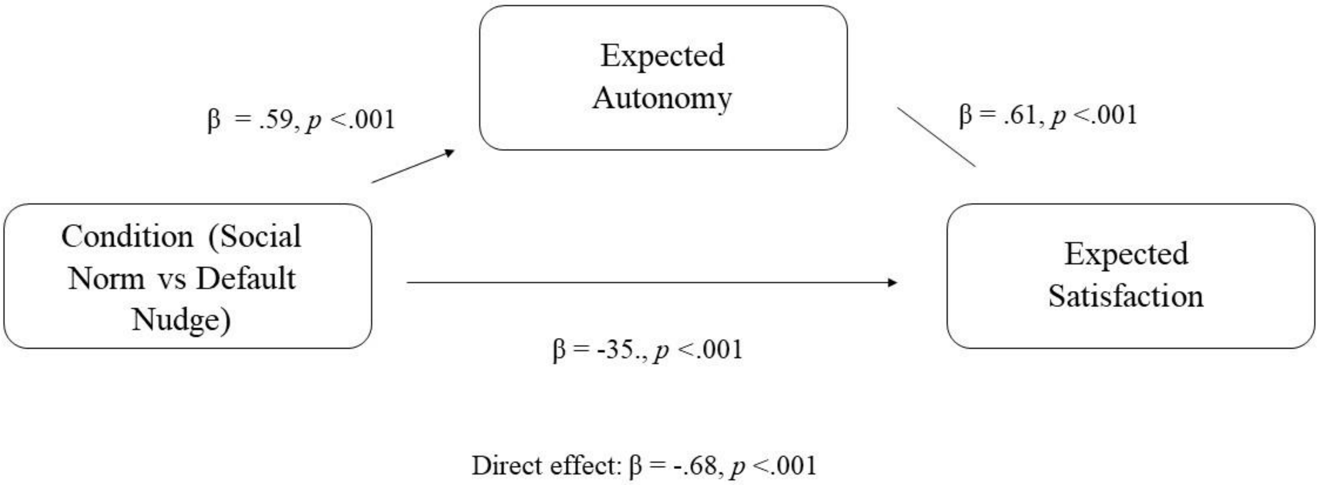


*Figure 10* Mediation of Expected Autonomy of the Effect of Condition (social norm nudge vs default nudge condition) on Expected Satisfaction in Study 3.
