# Supplementary material for: And How Would That Make You Feel? How People Expect Nudges to Influence Their Sense of Autonomy
Source: Front Psychol. 2020 Dec 11;11:607894. doi: 10.3389/fpsyg.2020.607894 (PMC7759476; doi:10.3389/fpsyg.2020.607894)
Supplement: Supplementary file 2 [file Data_Sheet_2.PDF]

And How Would That Make You Feel?
How People Expect Nudges to Influence Their Sense of Autonomy

List of amenities, as seen by the control condition in Study 1.

Green Amenities

- [ ] Energy Star furnace and air conditioner
- [ ] Tankless water heater
- [ ] Programmable thermostat
- [ ] Storm windows and doors
- [ ] Airflow-adjusting ceiling fans
- [ ] UV-filter film on windows
- [ ] Energy-efficient dishwasher and refrigerator
- [ ] Compact fluorescent light bulbs
- [ ] Energy-efficient washer and dryer
- [ ] Dimmer switches for indoor lighting
- [ ] Low-flow toilets
- [ ] Solar-powered outdoor lighting
- [ ] Low-flow faucets and shower heads
- [ ] Motion sensors for outdoor lighting

List of amenities, as seen by the default nudge condition in Study 1.

Green Amenities

- [x] Energy Star furnace and air conditioner
- [x] Tankless water heater
- [x] Programmable thermostat
- [x] Storm windows and doors
- [x] Airflow-adjusting ceiling fans
- [x] UV-filter film on windows
- [x] Energy-efficient dishwasher and refrigerator
- [x] Compact fluorescent light bulbs
- [x] Energy-efficient washer and dryer
- [x] Dimmer switches for indoor lighting
- [x] Low-flow toilets
- [x] Solar-powered outdoor lighting
- [x] Low-flow faucets and shower heads
- [x] Motion sensors for outdoor lighting

List of amenities, as seen by the direct persuasion condition in Study 1.

Green Amenities
Please think of the environment and select as many amenities as possible!

- ☐ Energy Star furnace and air conditioner
- ☐ Tankless water heater
- ☐ Programmable thermostat
- ☐ Storm windows and doors
- ☐ Airflow-adjusting ceiling fans
- ☐ UV-filter film on windows
- ☐ Energy-efficient dishwasher and refrigerator
- ☐ Compact fluorescent light bulbs
- ☐ Energy-efficient washer and dryer
- ☐ Dimmer switches for indoor lighting
- ☐ Low-flow toilets
- ☐ Solar-powered outdoor lighting
- ☐ Low-flow faucets and shower heads
- ☐ Motion sensors for outdoor lighting

Electricity scenario, as seen by the control condition in Study 2 and 3.

Imagine you are a student who just moved to a new flat in a new neighbourhood. Your monthly income is 800€. There are two possible contracts for you to choose from the local energy supplier. Please act as if real money was involved.

Usually, you spend your budget in the following way:
- Flat and utility bill: 330
- Alimentation: 160
- Clothing: 50
- Study materials: 30
- Transportation: 75
- Insurance and Medicine: 60
- Communication: 35
- Leisure: 60

Choose one of the following contracts:

- Contract A: 100% conventional energy, priced at 30€ per month.
- Contract B: 50% reneqable energy / 50% conventional energy, priced at 45€ per month.

Electricity scenario, as seen by the default nudge condition in Study 2 and 3.

Imagine you are a student who just moved to a new flat in a new neighbourhood. Your monthly income is 800€. There are two possible contracts for you to choose from the local energy supplier. Please act as if real money was involved.

Usually, you spend your budget in the following way:
- Flat and utility bill: 330
- Alimentation: 160
- Clothing: 50
- Study materials: 30
- Transportation: 75
- Insurance and Medicine: 60
- Communication: 35
- Leisure: 60

Choose one of the following contracts:

- ○ Contract A: 100% conventional energy, priced at 30€ per month.
- ◉ Contract B: 50% renewable energy / 50% conventional energy, priced at 45€ per month.

Electricity scenario, as seen by the direct persuasion condition in Study 2and 3.

Imagine you are a student who just moved to a new flat in a new neighbourhood. Your monthly income is 800€. There are two possible contracts for you to choose from the local energy supplier. Please act as if real money was involved.

Usually, you spend your budget in the following way:
- Flat and utility bill: 330
- Alimentation: 160
- Clothing: 50
- Study materials: 30
- Transportation: 75
- Insurance and Medicine: 60
- Communication: 35
- Leisure: 60

Please think of the environment and select the contract consisting of 50% renewable energy, if possible.

Choose one of the following contracts:

- Contract A: 100% conventional energy, priced at 30€ per month.
- Contract B: 50% renewable energy / 50% conventional energy, priced at 45€ per month.

Electricity scenario, as seen by the social norm nudge condition in Study 2 and 3.

Imagine you are a student who just moved to a new flat in a new neighbourhood. Your monthly income is 800€. There are two possible contracts for you to choose from the local energy supplier. Please act as if real money was involved.

Usually, you spend your budget in the following way:
- Flat and utility bill: 330
- Alimentation: 160
- Clothing: 50
- Study materials: 30
- Transportation: 75
- Insurance and Medicine: 60
- Communication: 35
- Leisure: 60

From your local energy provider you receive the information that the majority of your neighbours uses an energy mix that features 50% renewable energy.

Choose one of the following contracts:

- Contract A: 100% conventional energy, priced at 30€ per month.
- Contract B: 50% renewable energy / 50% conventional energy, priced at 45€ per month.
